# The CDK9 inhibitor enitociclib overcomes resistance to BTK inhibition and CAR-T therapy in mantle cell lymphoma

**DOI:** 10.1186/s40364-024-00589-7

**Published:** 2024-06-18

**Authors:** Vivian Jiang, William Lee, Tianci Zhang, Alexa Jordan, Fangfang Yan, Qingsong Cai, Joseph McIntosh, Jovanny Vargas, Yang Liu, Michael Wang

**Affiliations:** 1 Department of Lymphoma and Myeloma, The University of Texas MD Anderson Cancer Center, 1515 Holcombe Blvd, 77030 Houston, TX USA; 2 Department of Stem Cell Transplantation and Cellular Therapy, The University of Texas MD Anderson Cancer Center, Houston, TX USA

**Keywords:** CDK9, Mantle cell lymphoma, Enitociclib, VIP152, MYC, MCL-1, Relapse, Therapeutic resistance, BTK inhibitors, CAR-T therapy.

## Abstract

Inhibitors of Bruton’s tyrosine kinase (BTKi) and chimeric antigen receptor T-cell (CAR-T) therapy targeting CD19 are paradigm-shifting advances in treating patients with aggressive mantle cell lymphoma (MCL). However, clinical relapses following BTKi and CD19-directed CAR-T treatments are a fast-growing medical challenge. Development of novel therapies to overcome BTKi resistance (BTKi-R) and BTKi-CAR-T dual resistance (Dual-R) are urgently needed. Our single-cell RNA sequencing data revealed major transcriptomic reprogramming, with great enrichment of MYC-targets evolving as resistance to these therapies developed. Interestingly, cyclin-dependent kinase 9 (CDK9), a critical component of the positive transcription elongation factor-b complex, was among the top upregulated genes in Dual-R vs. BTKi-R samples. We therefore hypothesized that targeting CDK9 may turn off MYC-driven tumor survival and drug resistance. Enitociclib (formerly VIP152) is a selective CDK9 inhibitor whose potency against MCL has not been assessed. In this study, we found that enitociclib was highly potent in targeting lymphoma cells, with the half-maximal inhibitory concentration (IC_50_) ranging from 32 to 172 nM in MCL and diffuse large B-cell lymphoma cell lines. It inhibited CDK9 phosphorylation and downstream events including *de novo* synthesis of the short-lived proteins c-MYC, MCL-1, and cyclin D1, and induced apoptosis in a caspase-3-dependent manner. Enitociclib potently inhibited in vivo tumor growth of cell line-derived and patient-derived xenografts having therapeutic resistance. Our data demonstrate the potency of enitociclib in overcoming therapeutic resistance in MCL models and provide evidence in favor of its clinical investigation.

**Supplementary Information:**

The online version contains supplementary material available at 10.1186/s40364-024-00589-7.

## To the editor

**Fig. 1:**
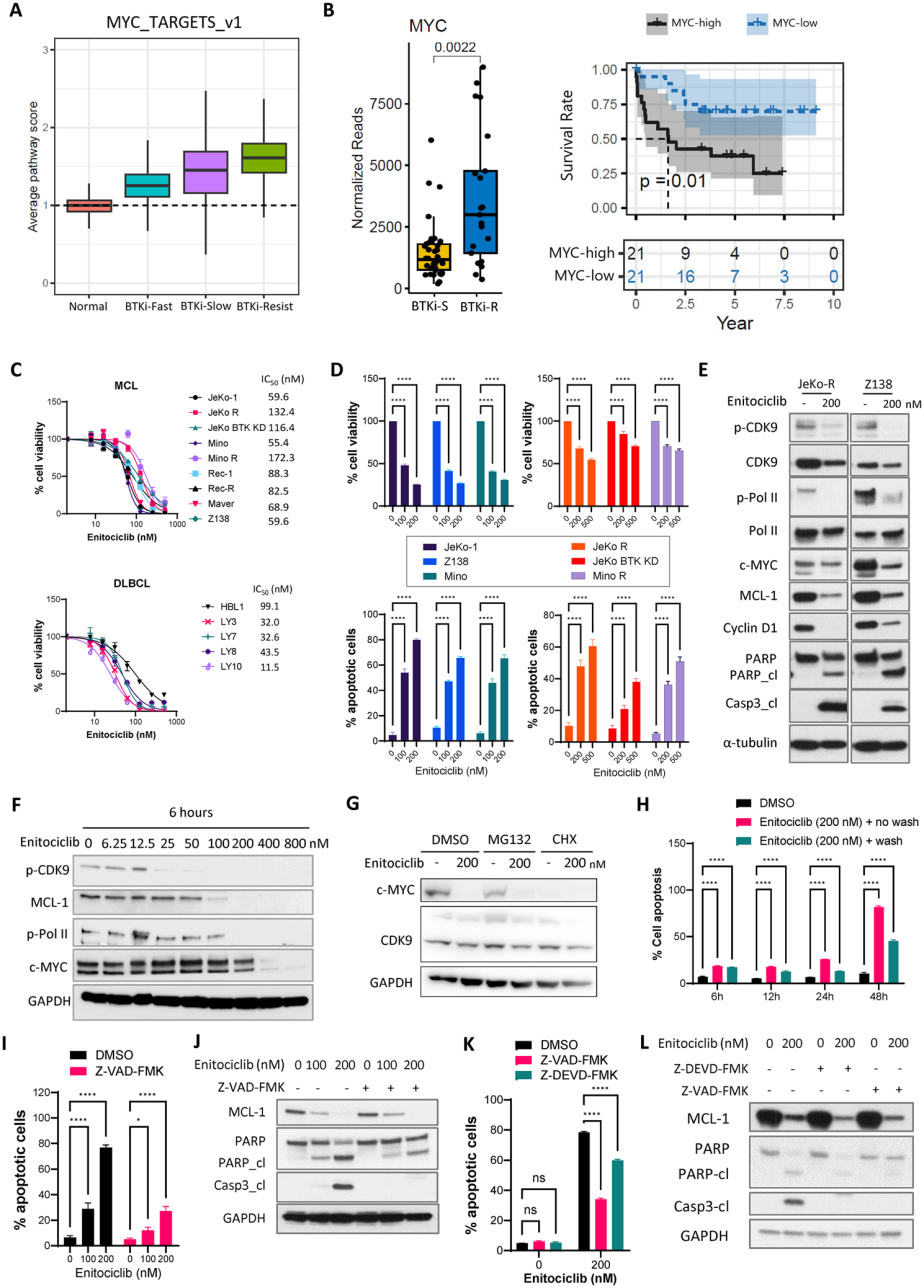
Targeting CDK9 with the specific inhibitor enitociclib potently inhibited lymphoma cell growth by suppressing *de novo* expression of short-lived proteins and inducing apoptosis. **(A)** MYC-TARGETS-v1 was progressively enriched in BTKi-fast responders (-Fast), -slow responders (-Slow) and non-responders (-Resist) based on GSEA analysis of single-cell RNA-seq data from MCL patient samples. **(B)** MYC mRNA expression was higher in BTKi-resistant (BTKi-R) than BTKi-sensitive (BTKi-S) MCL cells (left panel), and its high expression correlated with poor patient survival (right panel). **(C)** Cell viability assay assessing the in vitro efficacy of enitociclib in 9 MCL cell lines (top panel) and 5 DLBCL cell lines (bottom panel). The IC50 is presented to the right of each cell line. **(D)** Enitociclib at the indicated concentrations inhibited cell viability and induced apoptosis in a dose-dependent manner in MCL cells after 24 hr of treatment. **(E)** Western blots show that enitociclib inhibited CDK9 phosphorylation and Pol II phosphorylation at Ser 2, reduced expression of c-MYC, MCL-1, and cyclin D1, and induced cleavage of PARP and caspase-3 in JeKo-R and Z138 cells. **(F)** Enitociclib dose-dependently suppressed CDK9 phosphorylation, Pol II phosphorylation, and expression of c-MYC and MCL-1 by 6 hr after treatment in JeKo-R cells. **(G)** Pretreatment of cycloheximide (CHX, 50?g/ml) for 1 hour diminished c-MYC expression, while pretreatment of MG132 (10?M) failed to rescue enitociclib (200 nM)-induced c-MYC downregulation in JeKo-R cells. **(H)** Enitociclib (200 nM) induced apoptosis at 24 and 48 hr even when the cells were treated with enitociclib for only the first 6 hr in JeKo-R cells. **(I-J)** Pan-caspase inhibitor Z-VAD-FMK (10?M) blocked enitociclib (200 nM)-induced apoptosis by cell apoptosis assay **(I)** and western blot **(J)** in JeKo-R cells. **(K-L)** Caspase-3-specific inhibitor Z-DEAD-FMK (20?M) rescued enitociclib (200 nM)-induced apoptosis by cell apoptosis assay **(K)** and western blot **(L)** in JeKo-R cells. *, p < 0.05; **, p < 0.01; ***, p < 0.001; ****, p < 0.0001

Mantle cell lymphoma [1] is a very aggressive subtype of non-Hodgkin lymphoma. There have been paradigm-shifting therapeutic advances in the last decade, including BTKi therapies (ibrutinib, acalabrutinib, zanubrutinib, and pirtobrutinib) and anti-CD19 CAR-T therapy [2–6]. However, therapeutic relapse frequently occurs, and there is a rising need to prevent or overcome resistance in patients who relapse. Our single-cell RNA sequencing data showed that MYC targets were progressively enriched with BTKi resistance (Fig. 1A). *MYC* mRNA expression was upregulated in BTKi-R compared to BTKi-sensitive (BTKi-S) cells, and its high expression correlated with poor patient survival in our patient cohort (*p* = 0.037) (Fig. 1B) and two independent cohorts [7, 8] (*p* = 0.0032 and 0.0027, respectively) (Supplementary Figure S1 A-B). Moreover, cyclin-dependent kinase 9 (CDK9) was among the top upregulated genes in Dual-R samples vs. solely BTKi-R samples [9]. Therefore, we targeted the transcription gatekeeper CDK9 to see if that approach could overcome therapeutic resistance. CDK9 inhibition by small molecules such as AZD4573 induces acute loss of short-lived mRNA and proteins, including c-MYC and MCL-1 [10]. Enitociclib is a selective and potent CDK9 inhibitor with a better safety profile than AZD4573 [11]; however, its potency in treating MCL and whether it overcomes therapeutic resistance is not known.

Enitociclib was highly potent in primary MCL cells, MCL cell lines, and diffuse large B-cell lymphoma (DLBCL) cell lines, with an IC_50_ of 32–172 nM (Fig. 1C and Supplementary Figure S2 A). Enitociclib inhibited cell viability in a dose- and time-dependent manner by robustly inducing apoptosis (Fig. 1D and Supplementary Figure S2 B-C and S3A-B). Upon treatment with enitociclib for 6 h, CDK9 phosphorylation was markedly inhibited in both JeKo-R cells with *acquired* BTKi-resistance and Z138 cells with *primary* BTKi-resistance (Fig. 1E). Correspondingly, CDK9 downstream signaling events, including phosphorylation of RNA polymerase II (Pol II) at Ser 2, were also downregulated, along with reduced expression of the short-lived proteins c-MYC, MCL-1, and cyclin D1 (Fig. 1E). These changes were dose-dependent in JeKo-R cells (Fig. 1F). Similarly, cycloheximide (CHX), a translation elongation inhibitor, diminished c-MYC expression, while the proteasome inhibitor MG132 failed to rescue enitociclib-induced c-MYC downregulation (Fig. 1G and Supplementary Figure S4). These indicate that enitociclib blocks *de novo* gene expression of short-lived proteins but not protein degradation.

Additionally, the two apoptosis indicators, cleaved PARP (poly (ADP-ribose) polymerase) and cleaved caspase-3, were markedly upregulated upon CDK9 inhibition (Fig. 1E). Enitociclib treatment triggered apoptosis as early as 6 h and further augmented it at 24 and 48 h in JeKo-R cells (Fig. 1H). Enitociclib-triggered apoptosis was blocked by the pan-caspase inhibitor Z-VAD-FMK and by the specific caspase-3 inhibitor Z-VEAD-FMK in JeKo-R and JeKo-1 cells (Fig. 1I-L and Supplementary Figure S5 A-B). Together, these data demonstrate that targeting CDK9 with enitociclib triggered apoptosis in a caspase-3-dependent manner.

**Fig. 2:**
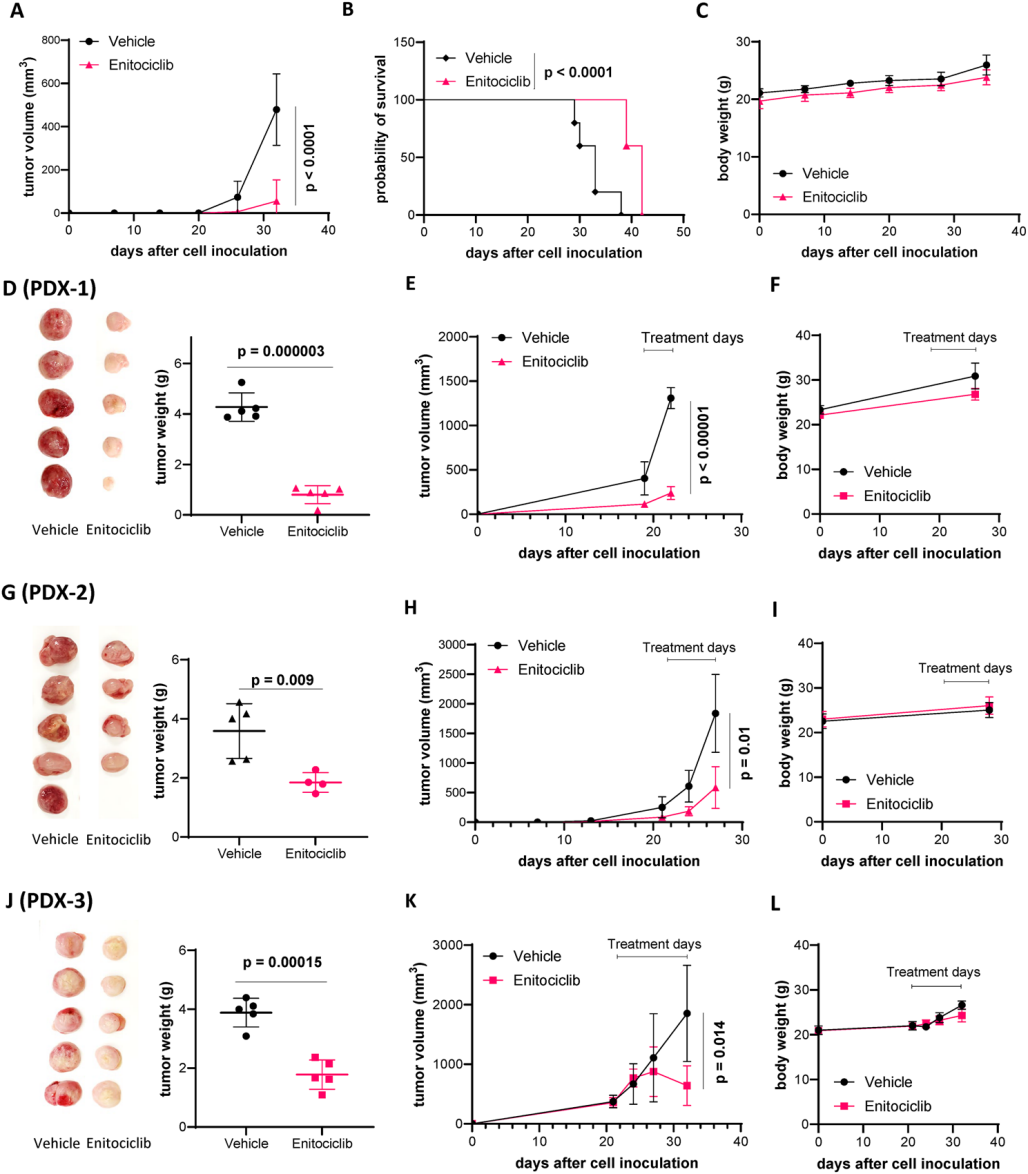
Enitociclib potently inhibited MCL cell growth in MCL cell line-derived xenografts (CDX) and patient-derived xenografts (PDX) models in mice. (**A-C**) Enitociclib (10 mg/kg, IV, twice a week) markedly inhibited tumor growth in JeKo-1 CDXs. Tumor volume (**A**), mouse survival (**B**), and mouse body weight (**C**) are plotted. Statistical significance is indicated in the graphs. (**D-L**) Enitociclib (10 mg/kg, IV, twice a week) effectively inhibited tumor growth in PDX models with ibrutinib resistance (**D-F**, PDX-1), ibrutinib-venetoclax dual resistance (**G-I**, PDX-2), or dual resistance to ibrutinib and CAR-T therapy (**J-L**, PDX-3). Mouse tumors were dissected, imaged, and weighed (**D, G,** and **J**). Tumor size (**E, H,** and **K**), and mouse body weight (**F, I, and L**) are plotted

To determine whether targeting CDK9 with enitociclib can effectively and safely overcome therapeutic resistance in MCL, we first tested its in vivo efficacy using JeKo-1 cell line-derived xenografts (CDXs). Enitociclib at 10 mg/kg (IV, twice a week) markedly inhibited the tumor growth of JeKo-1 CDXs in immunodeficient NSG (NOD.Cg-Prkdc^scid^Il2rg^tm1Wjl^/SzJ) mice (*p* < 0.0001) and correspondingly prolonged mouse survival (*p* < 0.0001) (Fig. 2A-B) without significant body weight loss (Fig. 2C) or other apparent adverse effect. To further address this, we established patient-derived xenograft (PDX) models from three patients with different types of therapeutic resistance: PDX-1 having BTKi resistance (Fig. 2D-F), PDX-2 having dual resistance to the BTKi ibrutinib and the Bcl-2 inhibitor venetoclax (Fig. 2G-I), and PDX-3 having dual BTKi-CAR-T resistance (Fig. 2J-L). Enitociclib efficaciously inhibited in vivo PDX growth of PDX-1 (*p* = 0.00015), PDX-2 (*p* = 0.009), and PDX-3 (*p* = 0.000003) without causing significant body weight loss (Fig. 2D-L).

Our findings showed that targeting CDK9 with its specific inhibitor enitociclib led to potent anti-lymphoma activity in vitro and in vivo. Enitociclib induced rapid CDK9 inhibition and a rapid decline in c-MYC, MCL-1, and cyclin D1 to robustly induce apoptosis, which is predominantly dependent on caspase-3 activation. Enitociclib also significantly impeded tumor growth in mouse CDX and PDX models. These data demonstrate that CDK9 is a promising target in MCL and may be utilized to overcome therapeutic resistance to BTKi and CART therapy in MCL. In a phase I dose-escalation trial, enitociclib was reported to be safe and effective in treating double-hit DLBCL patients [12]. Altogether, it highlights the targeting of CDK9 as a potentially effective regimen for treatment of advanced disease. Translational and mechanistic studies are ongoing to understand how targeting CDK9 can overcome therapeutic resistance in lymphoma.

### Electronic supplementary material

Below is the link to the electronic supplementary material.

Supplementary Material 1

Supplementary Material 2

Supplementary Material 3

Supplementary Material 4

Supplementary Material 5

Supplementary Material 6

## Data Availability

The single-cell RNA sequencing dataset and bulk RNA sequencing dataset have been deposited in in the European Genome-Phenome Archive (EGA) database under the accession codes EGAS00001005019 and EGAS00001003418. All other data generated or analyzed during this study are included in this published article and its supplementary information files.

## Abbreviations

BTK: Bruton’s tyrosine kinase
BTKi: BTK inhibitor
CAR-T: chimeric antigen receptor T cell
CDK9: cyclin-dependent kinase 9
CDX: cell line-derived xenograft
DLBCL: diffuse large B-cell lymphoma
BTKi-Fast: BTKi fast responder
BTKi-R: BTKi-resistant
BTKi-Resist: BTKi non-responders
BTKi-S: BTKi-sensitive
BTKi-Slow: BTKi slow responder
Dual-R: BTKi-CAR-T dual resistant
IC_50_: Half-maximal inhibitory concentration
MCL: mantle cell lymphoma
PDX: patient-derived xenograft
Pol II: RNA polymerase II
