# Supplementary figures and images for: The CDK9 inhibitor enitociclib overcomes resistance to BTK inhibition and CAR-T therapy in mantle cell lymphoma
Source: Biomark Res. 2024 Jun 18;12:62. doi: 10.1186/s40364-024-00589-7 (PMC11184686; doi:10.1186/s40364-024-00589-7)

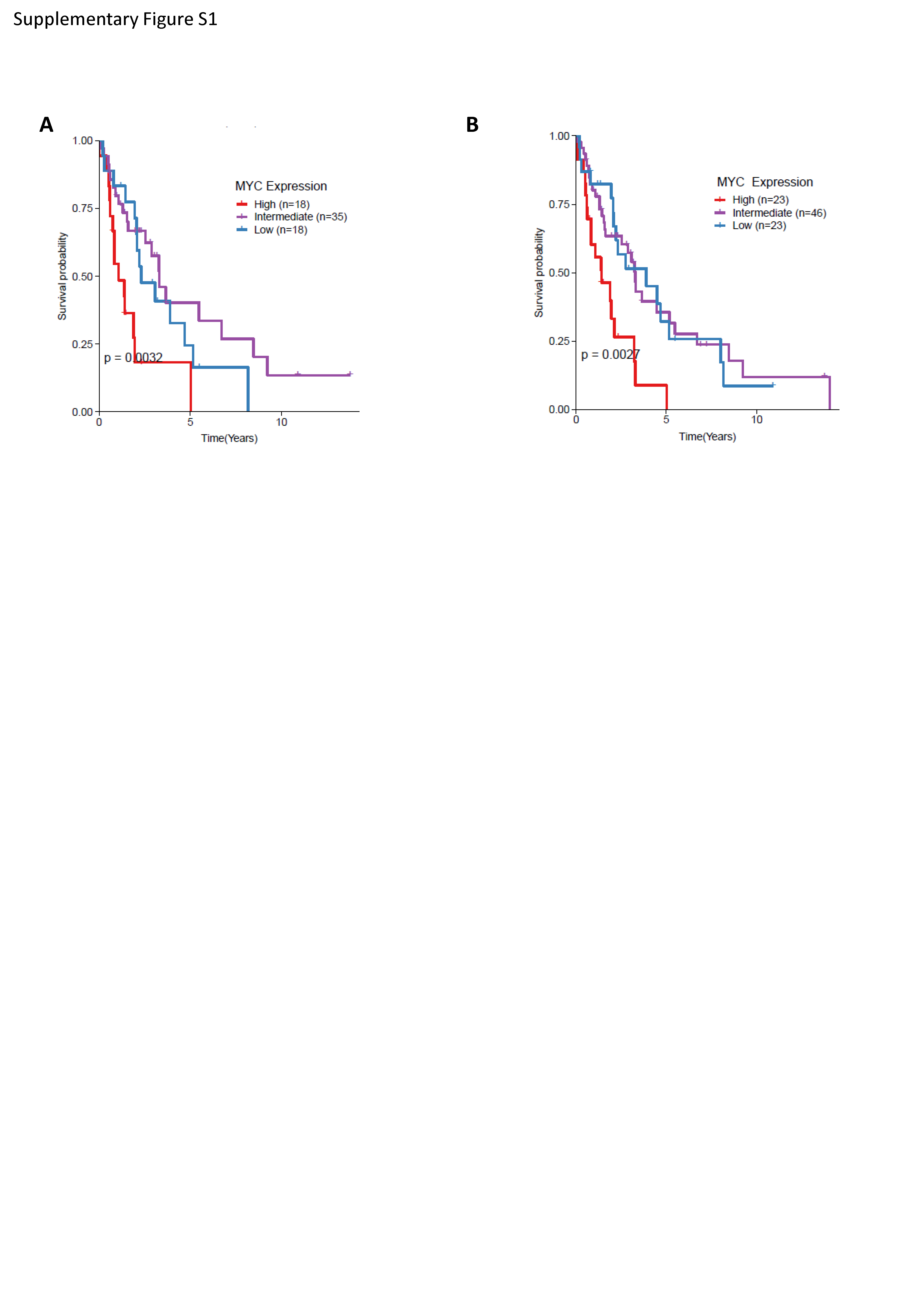

Supplement: Supplementary file 1 — Supplementary Material 1 [file 40364_2024_589_MOESM1_ESM.tif]

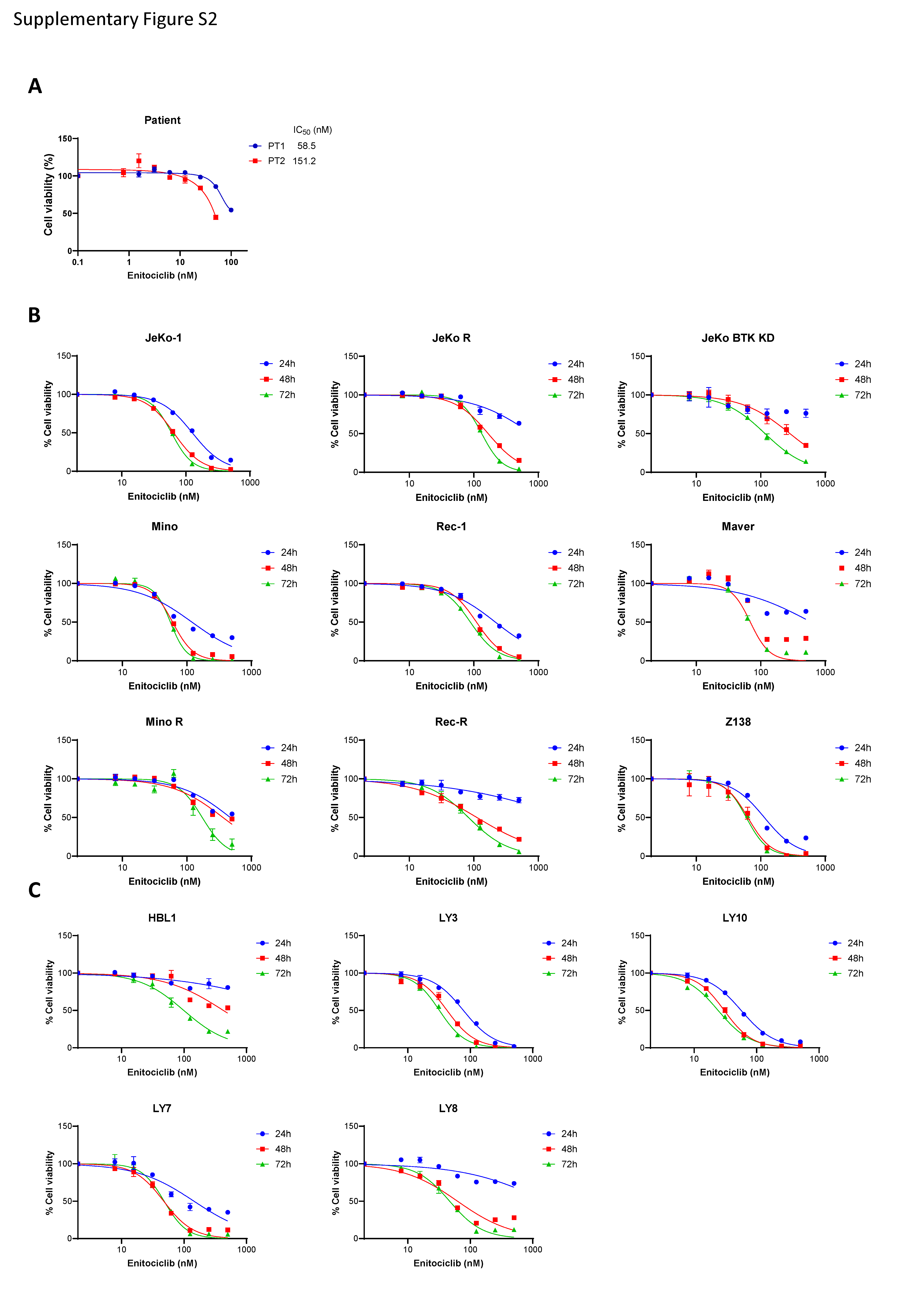

Supplement: Supplementary file 2 — Supplementary Material 2 [file 40364_2024_589_MOESM2_ESM.tif]

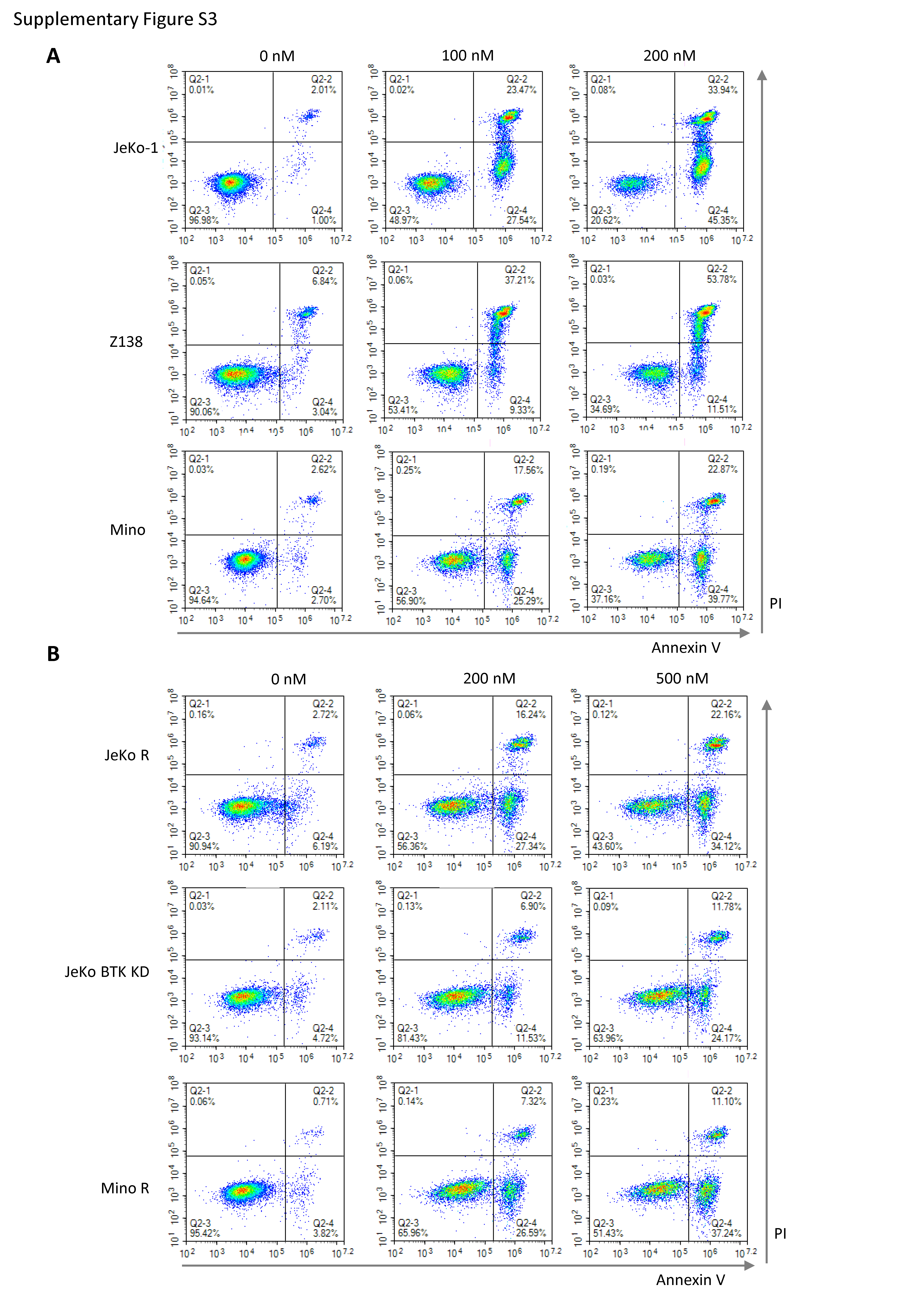

Supplement: Supplementary file 3 — Supplementary Material 3 [file 40364_2024_589_MOESM3_ESM.tif]

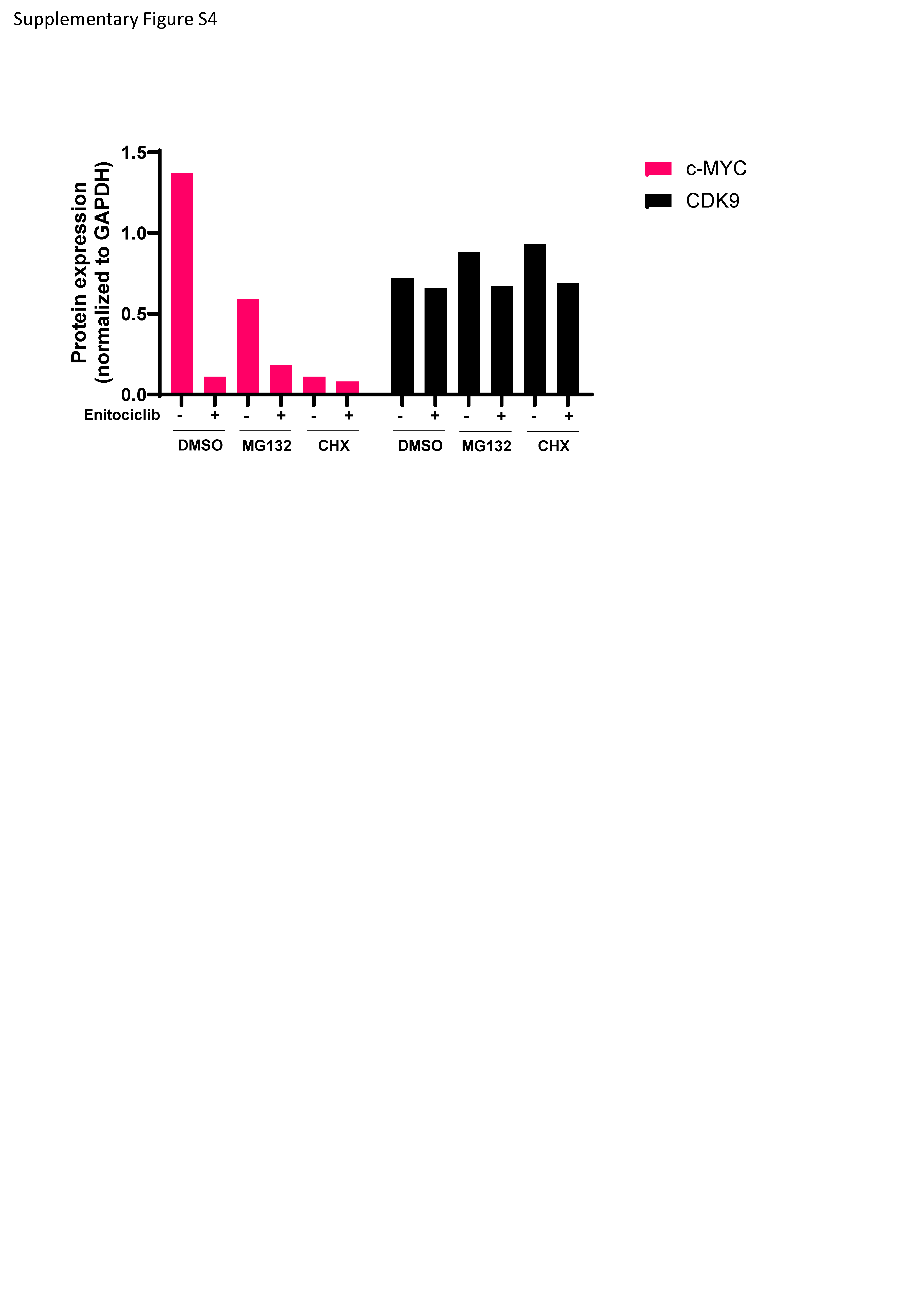

Supplement: Supplementary file 4 — Supplementary Material 4 [file 40364_2024_589_MOESM4_ESM.tif]

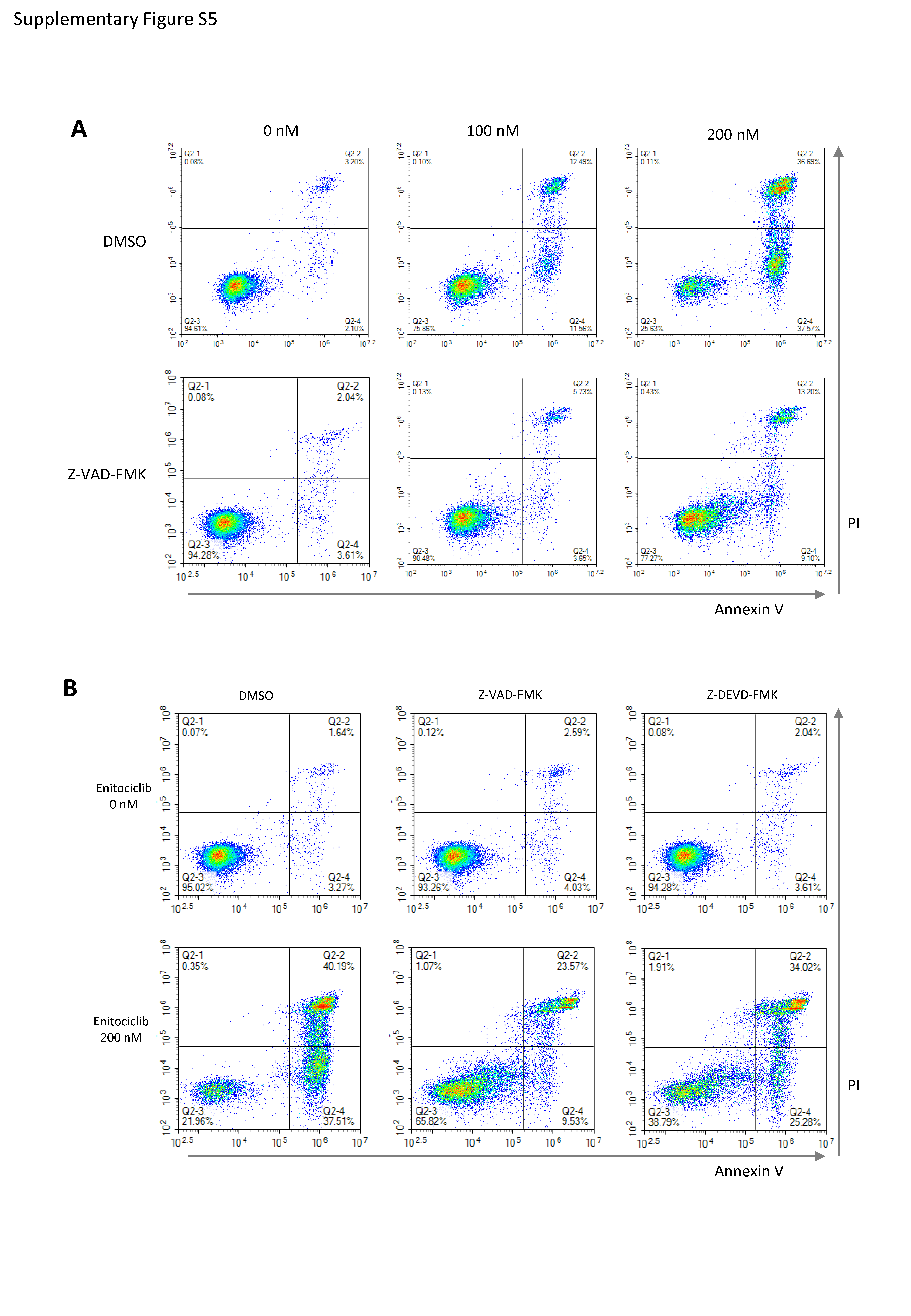

Supplement: Supplementary file 5 — Supplementary Material 5 [file 40364_2024_589_MOESM5_ESM.tif]
