# Supplementary material for: The CDK9 inhibitor enitociclib overcomes resistance to BTK inhibition and CAR-T therapy in mantle cell lymphoma
Source: Biomark Res. 2024 Jun 18;12:62. doi: 10.1186/s40364-024-00589-7 (PMC11184686; doi:10.1186/s40364-024-00589-7)
Supplement: Supplementary file 6 — Supplementary Material 6 [file 40364_2024_589_MOESM6_ESM.docx]

**Supplementary Figures**

Supplementary Figure S1


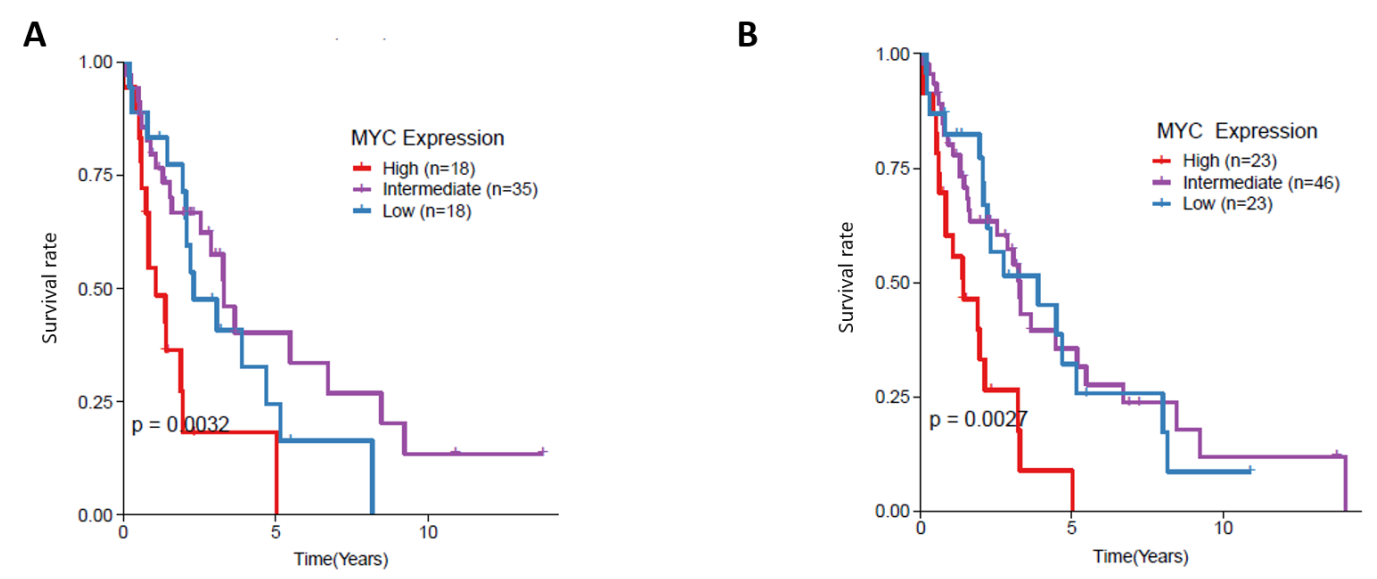


Supplementary Figure S2


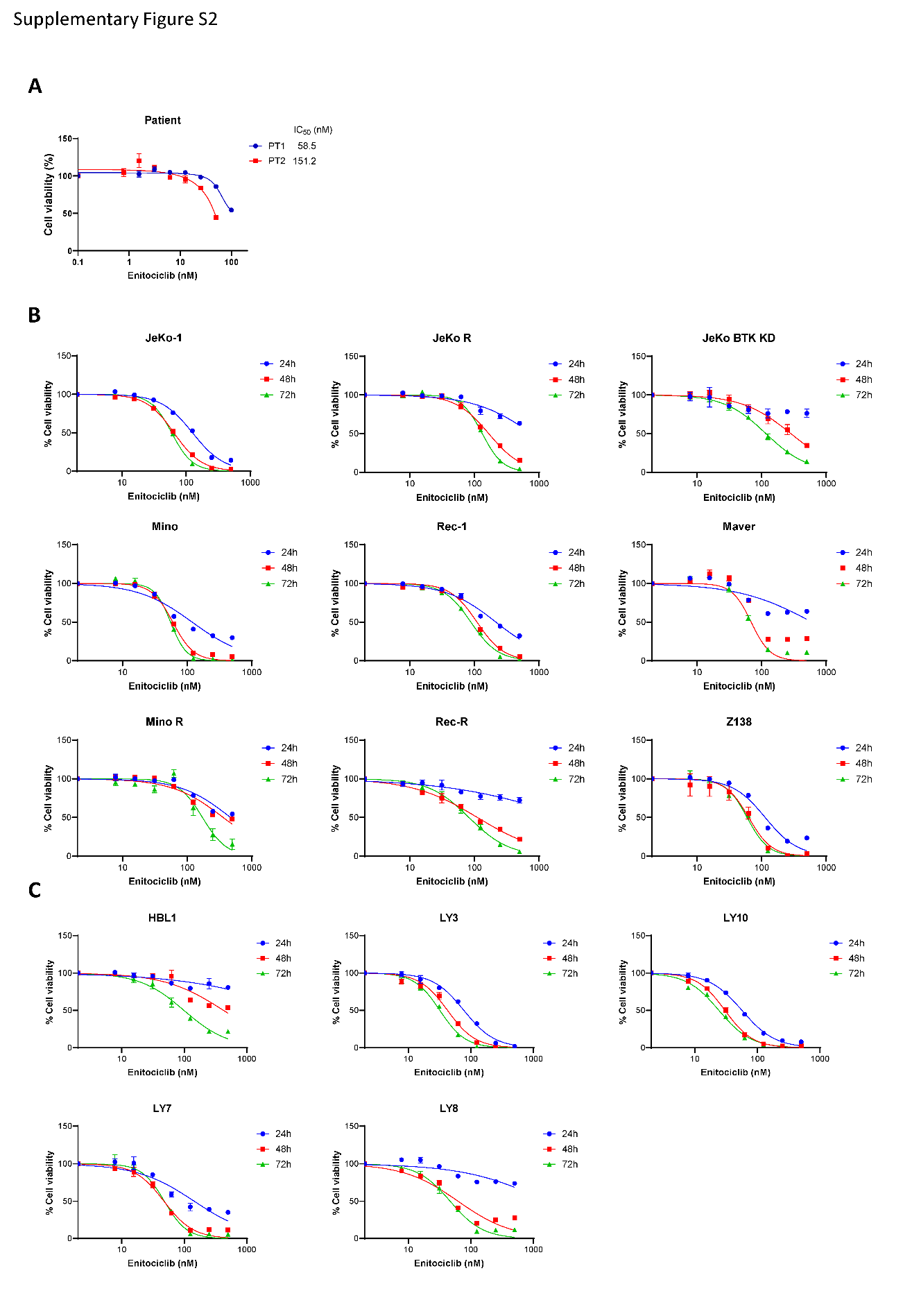


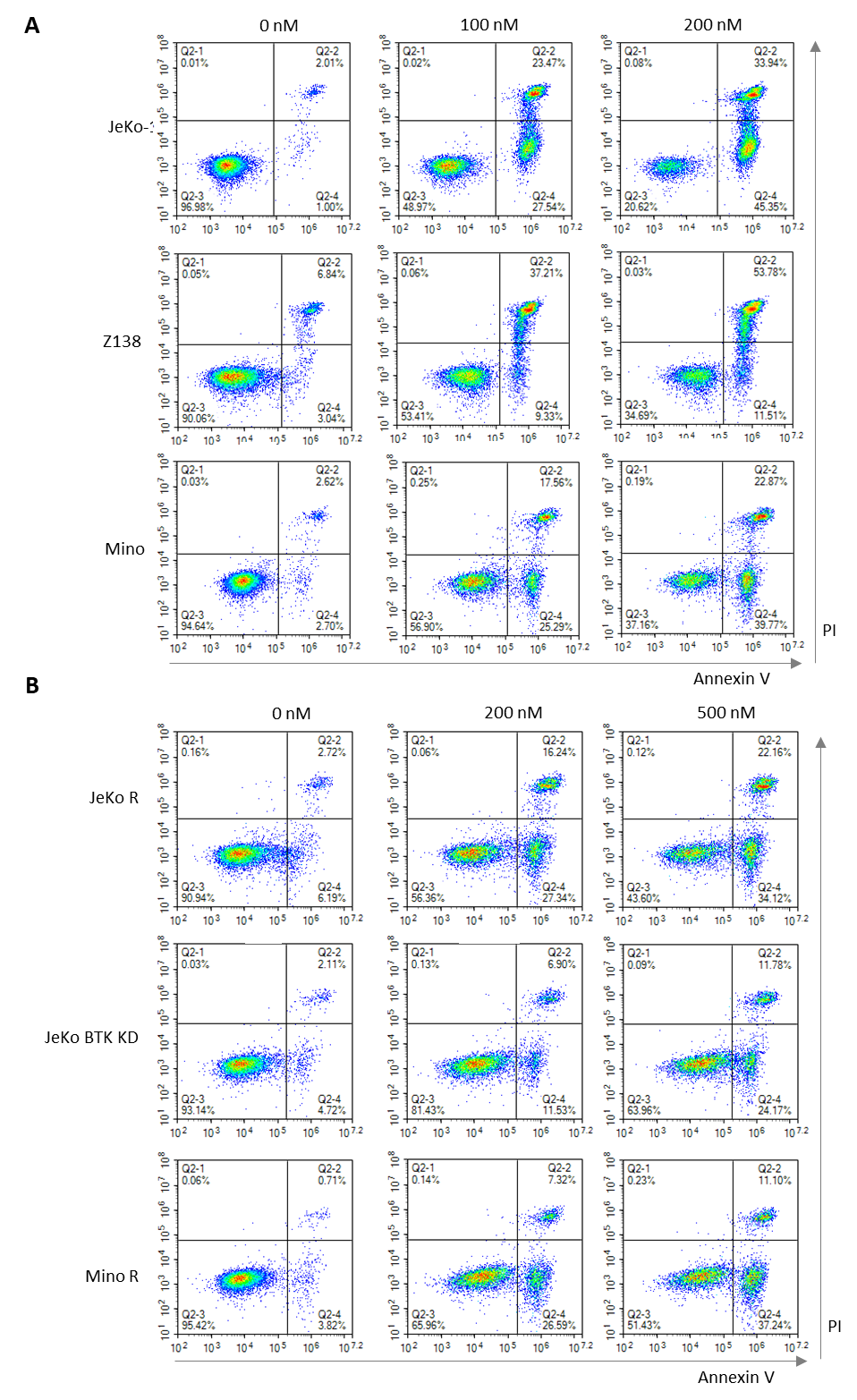
Supplementary Figure S3

Supplementary Figure S4

Supplementary Figure S5

**
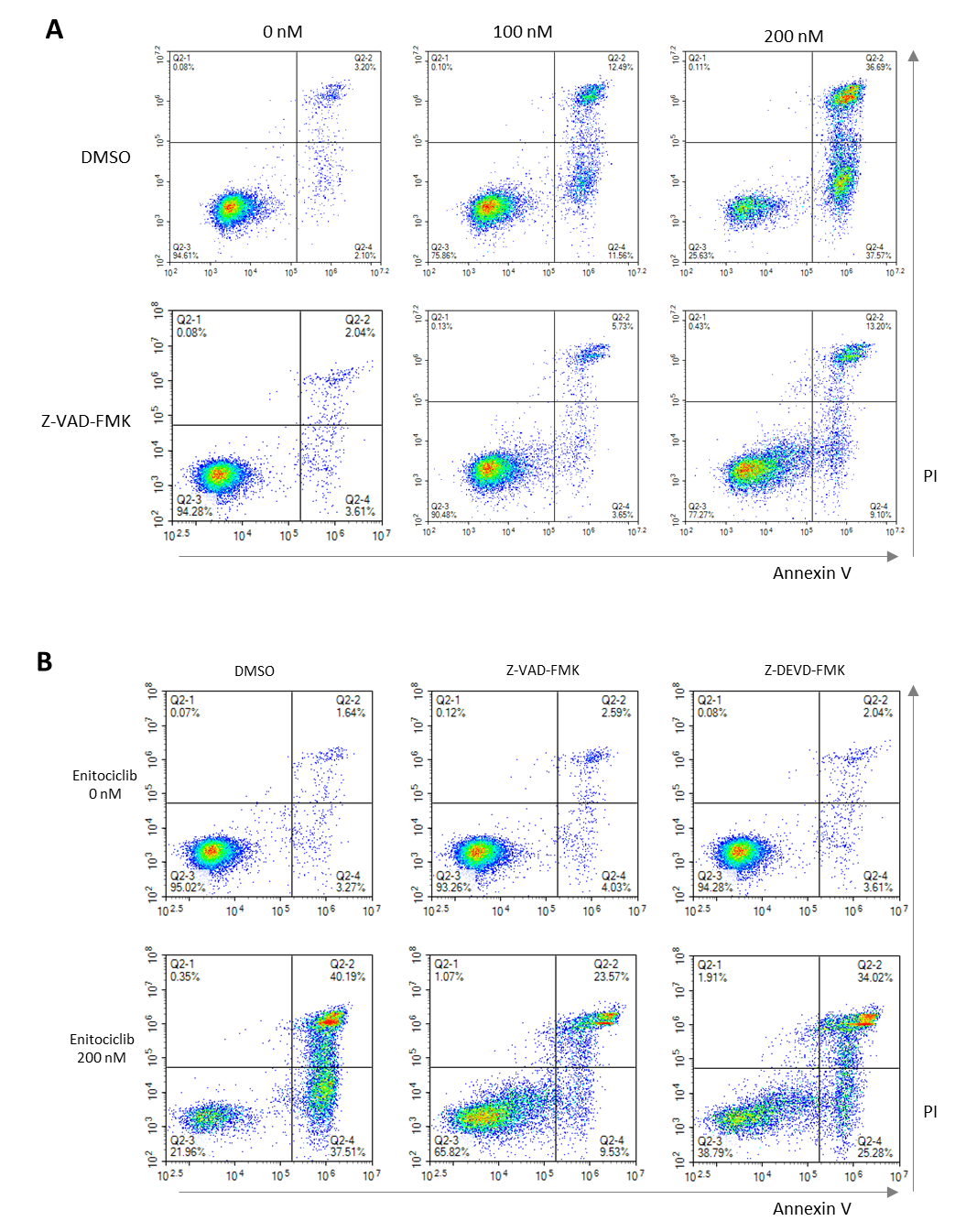
**

**Supplementary Figures**

**Supplementary Figure S1. High expression of *MYC* correlates with poor patient outcome in two independent patient cohorts.**

(**A-B**) High *MYC* mRNA expression correlates with poor patient survival in two published MCL patient cohorts (1, 2).

**Supplementary Figure S2. Enitociclib inhibited MCL and DLBCL cell growth in a dose- and time-dependent manner.**

(**A**) Cell viability assay shows enitociclib reduced viability of primary MCL cells isolated from two patients at 24 hours post treatment. PT1 with resistance to pirtobrutinib and PT2 with resistance to acalabrutinib. (**B-C**) Cell viability assay shows enitociclib reduced viability of MCL (**B**) and DLBCL(**C**) cell lines at 72 hours post treatment. Cell growth is dose-dependent (2-fold serial dilution from the top concentration of 500 nM) and time-dependent (24, 48, and 72 hr).

**Supplementary Figure S3. Enitociclib induced cell death via apoptosis.**

(**A-B**) Flow cytometry analysis shows that enitociclib induced apoptosis in JeKo-1, Z138 and Mino cells at 100 and 200 nM (**A**) and in JeKo R, JeKo BTK KD and Mino R cells at 200 and 500 nM (**B**) at 24 hr after treatment.

**Supplementary Figure S4. Enitociclib led to reduced de novo c-MYC expression.**

ImageJ was used to quantify the protein band intensity for each of the proteins detected by the western blots in Figure 1F. c-MYC and CDK9 expression was normalized to GAPDH expression.

**Supplementary Figure S5. Enitociclib-induced apoptosis is caspase-3-dependent.**

(**A**) Flow cytometry analysis shows that pan-caspase inhibitor Z-VAD-FMK (10 µM) inhibited enitociclib-induced apoptosis in JeKo-1 cells at 100 and 200 nM at 24 hr treatment. (**B**) Flow cytometry analysis shows that caspase-3 inhibitor Z-DEAD-FMK (20 µM) inhibited enitociclib-induced apoptosis in JeKo-1 cells at 200 nM at 24 hr after treatment.

**Supplemental methods**

*Cell viability and apoptosis assay*

The cell viability and apoptosis assay were performed as described previously (3). Briefly, MCL cell lines were plated at 20,000 per well in 96-well white plates and treated with enitociclib at the indicated concentrations. Cell viability was measured using CellTiter-Glo (Promega, Madison, WI, USA) at the indicated time post treatment. Annexin-V and PI staining with follow-up flow cytometry analysis was used to detect apoptosis post treatment with enitociclib at the indicated concentrations.

*Western blots*

The western blots were performed as described previously (4). Briefly, 5-10 million MCL cells were treated with enitociclib at the indicated concentrations with/without pretreatment of MG132 (10 µM), CHX (50 µg/ml), or caspase inhibitors Z-VAD_FMK (10 µM), or Z-DEVD-FMK (20 µM) for 1 hour. The cells were lysed in lysis buffer containing a protease inhibitor mixture (Roche Diagnostics) and subject to SDS-PAGE and Western blotting.

*In vivo drug efficacy testing in CDX and PDX models.*

In vivo drug efficacy testing in CDX and PDX models was performed as described previously (3). Briefly, MCL cells (2 million per mouse) or PDX cells (10 million per mouse) were subcutaneously injected into NSG (NOD.Cg-Prkdc^scid^Il2rg^tm1Wjl^/SzJ) mice. Once the tumors became palpable, the mice were treated with vehicle or enitociclib at 10 mg/kg (IV, twice a week). The tumor growth and mouse health conditions were monitored daily. The mice were euthanized at the end of the experiment, and the tumors were dissected and imaged. The tumor volume was calculated using the formula V = 0.5 × L × W^2^, where V is the tumor volume, L is the tumor length, and W is the tumor width.

*Statistics*

All experiments conducted in this study utilized at least 3 samples for in vitro experiments and 5 mice per group for in vivo experiments. Using a 2-sided *t*-test with a significance level of 0.05, each experiment had at least 80% power to detect an effect size of 3.1.

**Reference:**

1. Blenk S, Engelmann JC, Pinkert S, Weniger M, Schultz J, Rosenwald A, et al. Explorative data analysis of MCL reveals gene expression networks implicated in survival and prognosis supported by explorative CGH analysis. BMC Cancer. 2008;8:106.

2. Rosenwald A, Wright G, Wiestner A, Chan WC, Connors JM, Campo E, et al. The proliferation gene expression signature is a quantitative integrator of oncogenic events that predicts survival in mantle cell lymphoma. Cancer Cell. 2003;3(2):185-97.

3. Zhang S, Jiang VC, Han G, Hao D, Lian J, Liu Y, et al. Longitudinal single-cell profiling reveals molecular heterogeneity and tumor-immune evolution in refractory mantle cell lymphoma. Nat Commun. 2021;12(1):2877.

4. Jiang VC, Liu Y, Lian J, Huang S, Jordan A, Cai Q, et al. Cotargeting of BTK and MALT1 overcomes resistance to BTK inhibitors in mantle cell lymphoma. J Clin Invest. 2023;133(3).
